# Interactive effects of nitrogen and potassium on photosynthesis and photosynthetic nitrogen allocation of rice leaves

**DOI:** 10.1186/s12870-019-1894-8

**Published:** 2019-07-10

**Authors:** Wenfeng Hou, Merle Tränkner, Jianwei Lu, Jinyao Yan, Siyuan Huang, Tao Ren, Rihuan Cong, Xiaokun Li

**Affiliations:** 1 Key Laboratory of Arable Land Conservation (Middle and Lower Reaches of Yangtze River), Ministry of Agriculture/Microelement Research Center/College of Resources and Environment, Huazhong Agricultural University, Shizishan Street 1, Wuhan, 430070 China; 2 Department of Crop Sciences, Institute of Applied Plant Nutrition (IAPN), Georg-August-University Göttingen, Carl-Sprengel-Weg 1, 37075 Göttingen, Germany

**Keywords:** Nitrogen, Potassium, Photosynthesis, Photosynthetic nitrogen allocation, Photosynthetic nitrogen use efficiency, *Oryza sativa* L

## Abstract

**Background:**

Nitrogen (N) and potassium (K) are two important mineral nutrients in regulating leaf photosynthesis. Studying the interactive effects of N and K on regulating N allocation and photosynthesis (P_n_) of rice leaves will be of great significance for further increasing leaf P_n_, photosynthetic N use efficiency (PNUE) and grain yield. We measured the gas exchange of rice leaves in a field experiment and tested different kinds of leaf N based on N morphology and function, and calculated the interactive effects of N and K on N allocation and the PNUE.

**Results:**

Compared with N0 (0 kg N ha^− 1^) and K0 (0 kg K_2_O ha^− 1^) treatments, the P_n_ was increased by 17.1 and 12.2% with the supply of N and K. Compared with N0K0 (0 kg N and 0 kg K_2_O ha^− 1^), N0K120 (0 kg N and 120 kg K_2_O ha^− 1^) and N0K180 (0 kg N and 180 kg K_2_O ha^− 1^), N supply increased the absolute content of photosynthetic N (N_psn_) by 15.1, 15.5 and 10.5% on average, and the storage N (N_store_) was increased by 32.7, 64.9 and 72.7% on average. The relative content of N_psn_ was decreased by 5.6, 12.1 and 14.5%, while that of N_store_ was increased by 8.7, 27.8 and 33.8%. Supply of K promoted the transformation of N_store_ to N_psn_ despite the leaf N content (N_a_) was indeed decreased. Compared with N0K0, N180K0 (180 kg N and 0 kg K_2_O ha^− 1^) and N270K0 (270 kg N and 0 kg K_2_O ha^− 1^), K supply increased the relative content of N_psn_ by 17.7, 8.8 and 7.3%, and decreased the relative content of N_store_ by 24.2, 11.4 and 8.7% respectively.

**Conclusions:**

This study indicated the mechanism that K supply decreased the N_a_ but increased the N_psn_ content and then increased leaf P_n_ and PNUE from a new viewpoint of leaf N allocation. The supply of K promoted the transformation of N_store_ to N_psn_ and increased the PNUE. The decreased N_store_ mainly resulted from the decrease of non-protein N. Combined use of N and K could optimize leaf N allocation and maintain a high leaf N_psn_ content and PNUE.

**Electronic supplementary material:**

The online version of this article (10.1186/s12870-019-1894-8) contains supplementary material, which is available to authorized users.

## Background

Nitrogen (N) is one of the most important nutrients that limit the growth of plants. The economics of N use in photosynthesis has become a research hotspot in the past several decades and has continued to this day [1]. Leaves accumulated most of N in plant and as much as three quarters of leaf N was invested into photosynthetic apparatus, which was the largest N sink in plant [2–4]. It was reported that there was strong positive correlation between photosynthetic rate (P_n_) and leaf N content [5–8]. The leaf photosynthesis was largely controlled by the supply and demand of leaf N content. The ratio between P_n_ and leaf N content defines the photosynthetic N use efficiency (PNUE) as the amount of CO_2_ fixed by per unit of leaf N, which was varied as a function of leaf N content [4, 9]. There were different forms of N in leaves, like nitrates, amino acids and protein in soluble components, cell walls, membranes and other structures in insoluble components. Different species had different patterns of N allocation to various components contain N, and these differences caused the disparity in P_n_ and PNUE between species. Makino et al. [10] indicated that there were significant differences between C_3_ plants (such as rice) with C_4_ plants (such as maize) on leaf N allocation and PNUE. The soluble protein in maize (33%) was significantly lower than that in rice (50%), by contrast, the insoluble N in maize (53%) was appreciably higher than that in rice (37%) [10]. Takashima et al. [11] indicated that evergreen species had smaller N allocation to photosynthetic apparatus and smaller PNUE than that of deciduous species. Evergreen species allocated two-fold more N to SDS (3% sodium dodecyl sulfate)-soluble proteins than that of deciduous species, which indicated that evergreen species allocated more N to cell wall than deciduous species to maintain a long leaf lifespan. Onoda et al. [12] reported that plants germinated later had a higher PNUE than that germinated earlier, which was attributed to a larger N allocation to ribulose 1, 5-bisphosphate carboxylase/oxygenase (Rubisco). Early germinator leaves invested more N to cell wall to produce structurally tough leaves and maintain a long photosynthesis period, which was at the expense of lower allocation of N to Rubisco, lower P_n_ and lower PNUE. Ellsworth et al. [13] indicated that the leaf N allocated to Rubisco declined with the increase of structural N. A research on the N allocation of *Ageratina adenophora* showed that the invasive plants allocated 13.0% more leaf N to photosynthesis and had 24.4% higher P_n_ and 20.2% higher PNUE that that of native plants, and there was no significant differences between leaf total N content [14]. Also as expected, invasive plants allocated 45.2% lower cell wall protein content, 37.8% lower ratio of cell wall protein to leaf total protein, and 46.5% lower ratio of leaf N allocated to cell wall [14].

The variations in leaf N allocation was not only determined by the inherent characteristics but also influenced by the environment. Previous studies did some research on the N allocation for key photosynthetic enzymes and mainly focus on the environment factors, such as light condition, temperature, CO_2_ concentration and so on. Hikosaka and Terashima [15] developed a model to summarize the different roles of all photosynthetic components and predicted a highlight would promote the allocation of leaf N to Calvin cycle enzymes and electron carriers. Low light resulted in relatively even N allocation to light harvesting and carbon assimilation. The shade leaves had a 1:1 ratio of soluble protein and membrane-bound protein while sun leaves had 2–3 times more soluble protein than membrane-bound protein [16]. Xu et al. [17] built a complete N allocation model that simultaneously considers N allocation to light capture, electron transport, carboxylation, respiration and storage, and also including the different responses to the altered environment conditions such as CO_2_ concentration, temperature and radiation. The results indicated that the increase of temperature from 15 to 20 °C decreased about 10% of the N allocation to carboxylation. The increase of CO_2_ concentration from 370 to 570 μmol mol^− 1^ could decrease about 15% of the N allocated to carboxylation. The decrease of radiation from 800 to 400 μmol m^− 2^ s^− 1^ had small effect on N allocation to carboxylation for deciduous and evergreen trees but decreased about 10% of that for herbaceous plants. Meanwhile, the lower radiation decreased the storage N and as compensation, N allocated to light capture was increased.

As two major important macro-elements, the application of N and potassium (K) could significantly increase the P_n_ of rice leaves. Frak et al. [18] and Oguchi et al. [19] indicated that supply of N and N allocation could play important role in leaves photosynthetic acclimation exposed to increased irradiance. Niedz and Evens revealed that NH_4_^+^ and K^+^ exhibit strong synergistic blending on the growth of nonembryogenic and embryogenic tissue of sweet orange [20]. The leaf K content could be significantly increased for the supply of K while the leaf N content was significantly decreased at the same time [21]. However, the supply of K could further improve the P_n_, even though the leaf N content was significantly decreased [21]. Based on the previous studies on N allocation, we assumed that the supply of K could also affect the allocation of leaf N and did a field trial in 2016 to study the interactive effects of N and K on N allocation of rice leaves. To the best of our knowledge, few studies have been conducted on leaf N allocation with the interaction of N and K and the underlying physiological mechanism of N and K effects on P_n_ and PNUE remains unclear. The objectives of our study were as follows: (1) to assess the effects of N and K on the leaf P_n_ and PNUE; (2) to uncover the different effects of N and K on the allocation of leaf N; (3) to explain the phenomenon how does the supply of K increased the P_n_ while decreased the leaf N content from the angle of photosynthetic N allocation.

## Results

### Leaf morphological and physiological traits

The results indicated that leaf morphological and physiological traits including leaf dry mass and area, chlorophyll content, N content (N_a_) and K content (K_a_) were significantly influenced by the interaction effects of N and K (Table 1). Compared with N0 (0 kg N ha^− 1^) and K0 (0 kg K_2_O ha^− 1^) treatments, the application of N and K significantly increased the leaf dry mass per plant by 91.3 and 19.3% on average, and the leaf area per plant was increased by 77.9 and 36.6% on average, respectively. Compared with N0K0 (0 kg N and 0 kg K_2_O ha^− 1^), the leaf dry mass and leaf area per plant of N270K120 (270 kg N and 120 kg K_2_O ha^− 1^) was increased by 178.5 and 183.7% respectively, which had the maximum increase rate. The leaf chlorophyll content and N_a_ was significantly increased by 15.5 and 27.3% on average with the supply of N, while that was decreased by 15.0 and 7.0% on average for the supply of K. The leaf K_a_ was decreased by 16.1% with the supply of N, while that was increased by 14.6% for the supply of K.

**Table 1:** Interactive effect of nitrogen and potassium on leaf dry mass, leaf area, chlorophyll content, nitrogen content (N_a_) and potassium content (K_a_)

Treatments		Leaf dry mass g plant^−1^	Leaf area cm^2^ plant^− 1^	Chlorophyll mg m^− 2^	N_a_ g m^− 2^	K_a_ g m^− 2^
N0	K0	3.16 ± 0.18 bC^a^	602 ± 36 cB	146.9 ± 1.5 aB	1.14 ± 0.02 aC	1.05 ± 0.05 bA
	K120	4.52 ± 0.11 aC	859 ± 47 bC	125.2 ± 8.9 bB	1.02 ± 0.01 bC	1.10 ± 0.01 bA
	K180	4.86 ± 0.15 aB	942 ± 48 aB	118.1 ± 1.3 bB	1.01 ± 0.03 bC	1.17 ± 0.01 aA
N180	K0	5.04 ± 0.18 bB	1114 ± 65 cA	155.6 ± 6.1 aB	1.34 ± 0.04 aB	0.88 ± 0.02 cB
	K120	5.85 ± 0.17 aB	1297 ± 77 bB	143.6 ± 5.9 abA	1.27 ± 0.03 bB	0.96 ± 0.02 bB
	K180	6.11 ± 0.22 aA	1553 ± 132 aA	140.4 ± 6.4 bA	1.25 ± 0.10 bB	1.06 ± 0.04 aB
N270	K0	8.06 ± 0.21 bA	1220 ± 145 bA	175.8 ± 3.7 aA	1.45 ± 0.05 aA	0.77 ± 0.02 cC
	K120	8.80 ± 0.28 aA	1708 ± 72 aA	144.1 ± 2.8 bA	1.41 ± 0.02 abA	0.91 ± 0.01 bC
	K180	8.69 ± 0.39 abA	1660 ± 97 aA	141.5 ± 13.5 bA	1.36 ± 0.02 bA	0.99 ± 0.02 aC
N		**^b^	**	**	**	**
K		**	**	**	**	**
N × K		*^c^	**	*	*	*

^a^ Different small letters were used to show the significant differences between three K rates of the same N rate. Different large letters were used to show the significant differences between three N rates of the same K rate

^b^ ** means significant difference at *P* ≤ 0.01

^c^ * means significant difference at *P* ≤ 0.05, ns means no significant difference

### Photosynthetic rate

**Fig. 1:**
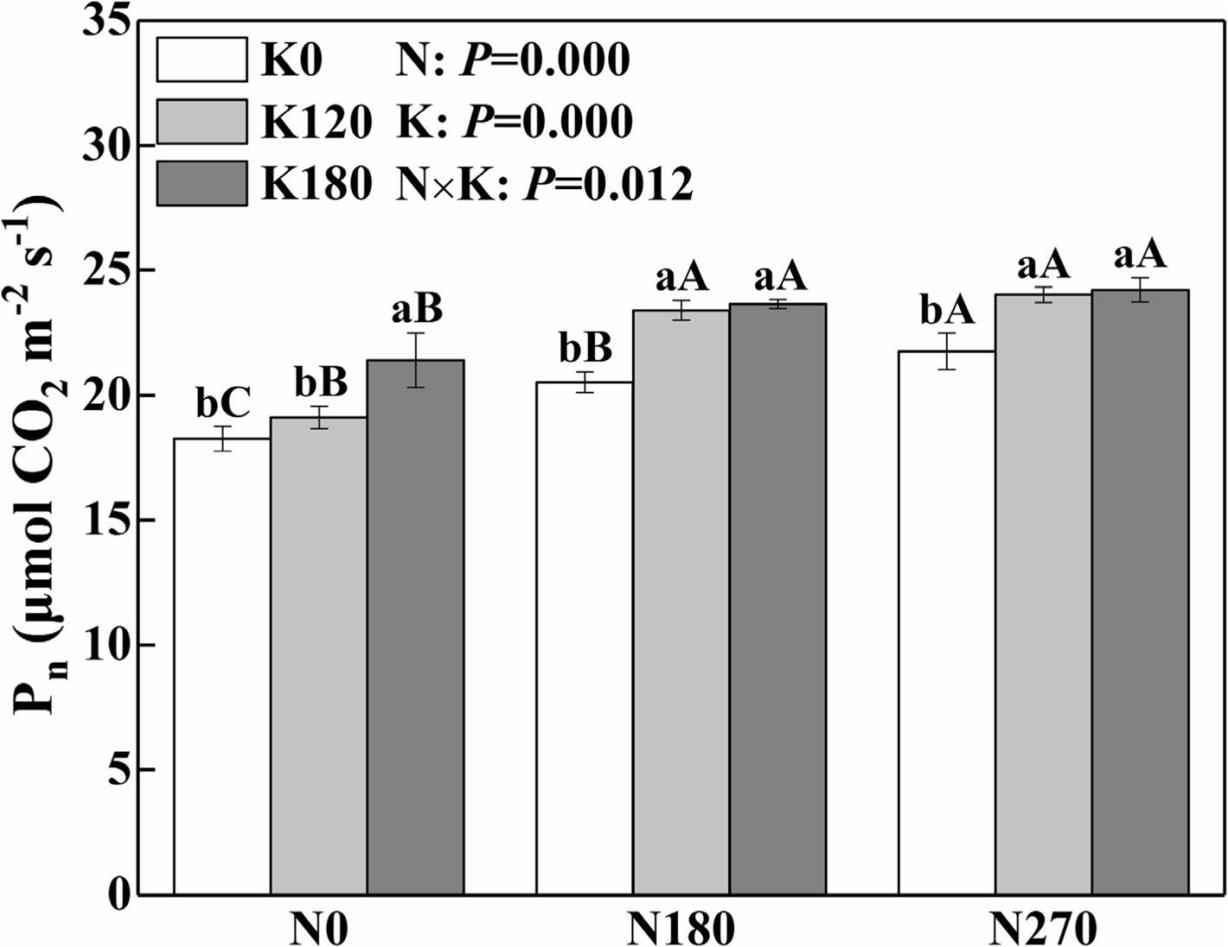
Interactive effects of nitrogen and potassium rates on photosynthesis rate (P_n_) of rice leaves

The leaf photosynthetic rate (P_n_) was significantly influenced by N, K and their interaction effects (Fig. 1). Compared with N0 and K0 treatments, the application of N and K significantly increased the P_n_ by 17.1 and 12.2% on average. Compared with N0K0, the P_n_ of N270K180 (270 kg N and 180 kg K_2_O ha^− 1^) was increased by 32.6%, which had the maximum increase rate.

### Nitrogen allocation by function

**Fig. 2:**
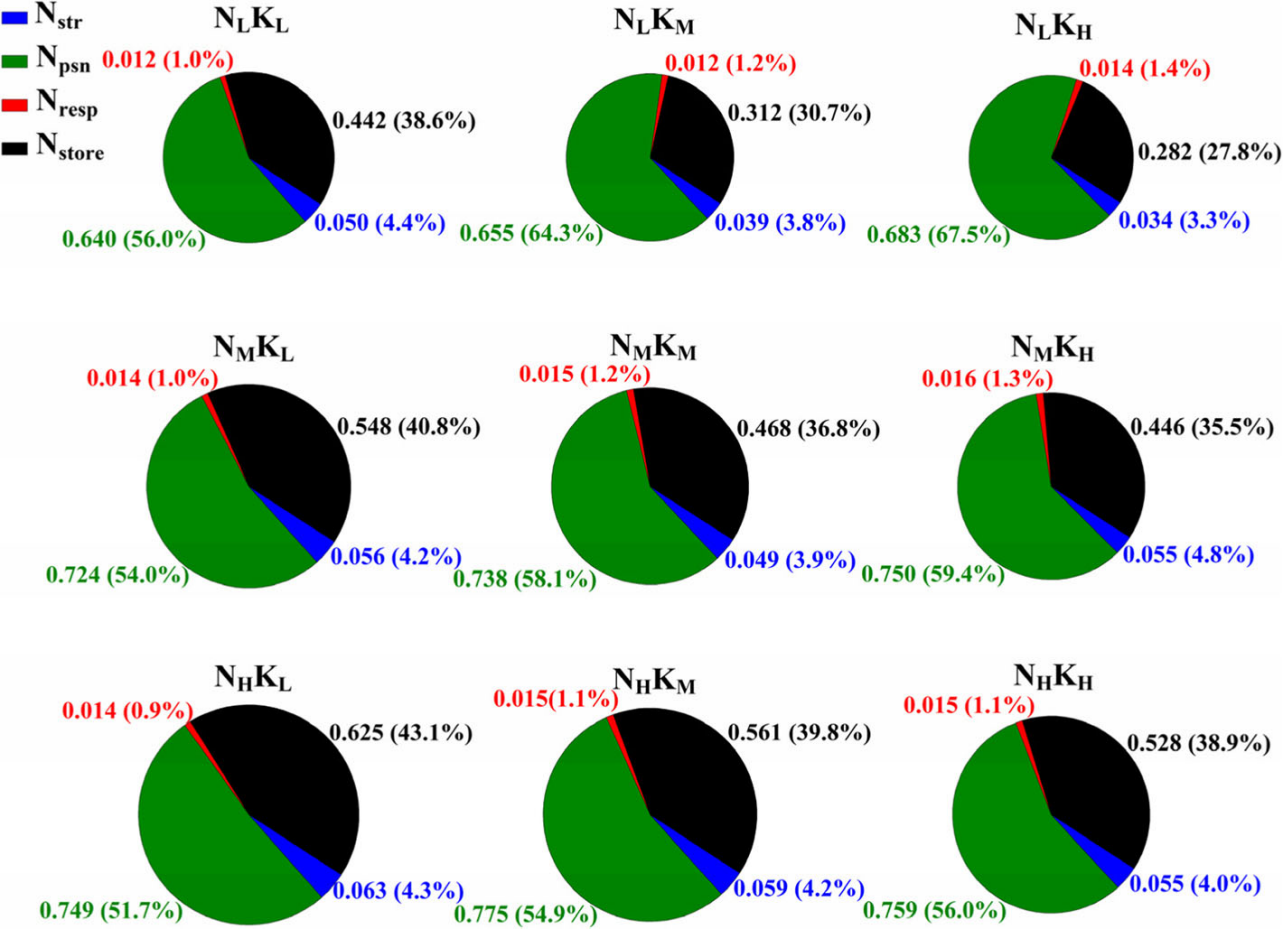
Interactive effects of nitrogen and potassium on leaf N allocation by morphology. N_str_ represents structural N, N_psn_ represents photosynthetic N, N_rep_ represents respiratory N, N_store_ represents storage N. The number out of the bracket represents the absolute N content (g m^− 2^) of different morphologies. The number in the bracket represents the relative content (%)

Interactive effects of N and K on leaf N allocation by function was shown in Fig. 2. The N_a_ was significantly increased with the supply of N while significantly decreased with the supply of K. Compared with N0 and K0 treatment, the N_a_ was increased by 27.5% for the supply of N and decreased by 6.8% for the supply of K. The results also indicated that the increase of N significantly increased the absolute content (g m^− 2^) (outside of the bracket) of photosynthetic N (N_psn_) and storage N (N_store_). However, the relative content (%) (in the bracket) of N_psn_ was decreased with the increase of N rate, while that of N_store_ was increased with the increase of N rate. Compared with N0K0, N0K120 (0 kg N and 120 kg K_2_O ha^− 1^) and N0K180 (0 kg N and 180 kg K_2_O ha^− 1^), the supply of N increased the absolute content of N_psn_ by 15.1, 15.5 and 10.5% on average, and that of N_store_ was increased by 32.7, 64.9 and 72.7% on average, respectively. The relative content of N_psn_ was decreased by 5.6, 12.1 and 14.5%, while that of N_store_ was increased by 8.7, 27.8 and 33.8%.

Both the absolute and relative content of N_psn_ were significantly increased with the supply of K. Compared with N0K0, N180K0 (180 kg N and 0 kg K_2_O ha^− 1^) and N270K0 (270 kg N and 0 kg K_2_O ha^− 1^), the supply of K increased the absolute content of N_psn_ by 4.5, 2.8 and 2.4%, and the relative content of N_psn_ was increased by 17.7, 8.8 and 7.3% on average, respectively. Both the absolute and relative content of N_store_ were significantly decreased with the supply of K. Compared with N0K0, N180K0 and N270K0, the supply of K decreased the absolute content of N_store_ by 32.8, 16.6 and 12.9%, and the relative content of N_store_ was decreased by 24.2, 11.4 and 8.7% on average, respectively.

**Fig. 3:**
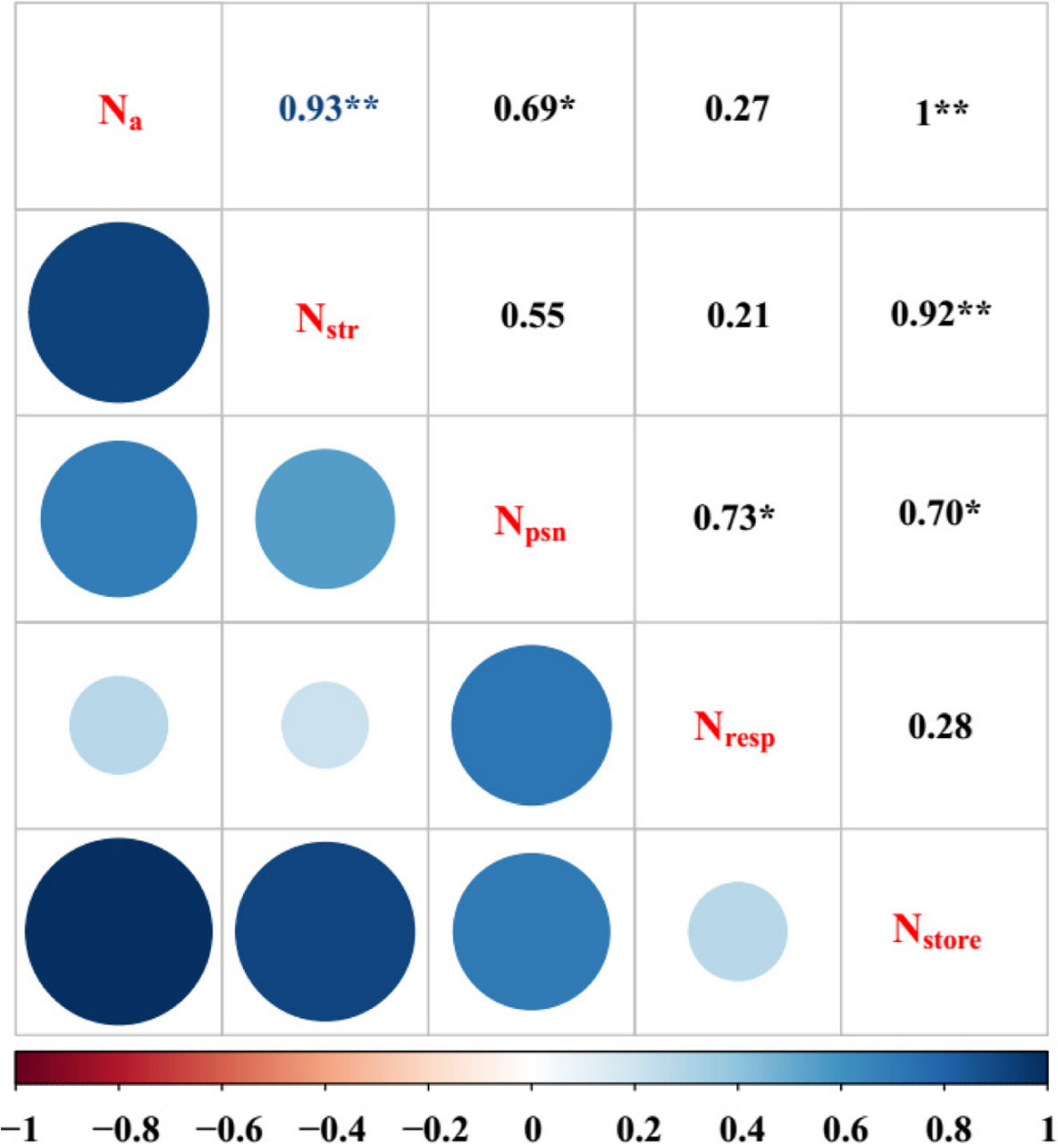
Correlation matrix between the different N morphologies with the N_a_. N_a_ represents the total leaf N content, N_str_ represents structural N, N_psn_ represents photosynthetic N, N_rep_ represents respiratory N, N_store_ represents stor*a*ge N

Correlation matrix between the different N morphologies with the N_a_ showed that N_store_ and N_psn_ had significant positive correlation with N_a_ (Fig. 3).

### Photosynthetic nitrogen allocation

**Fig. 4:**
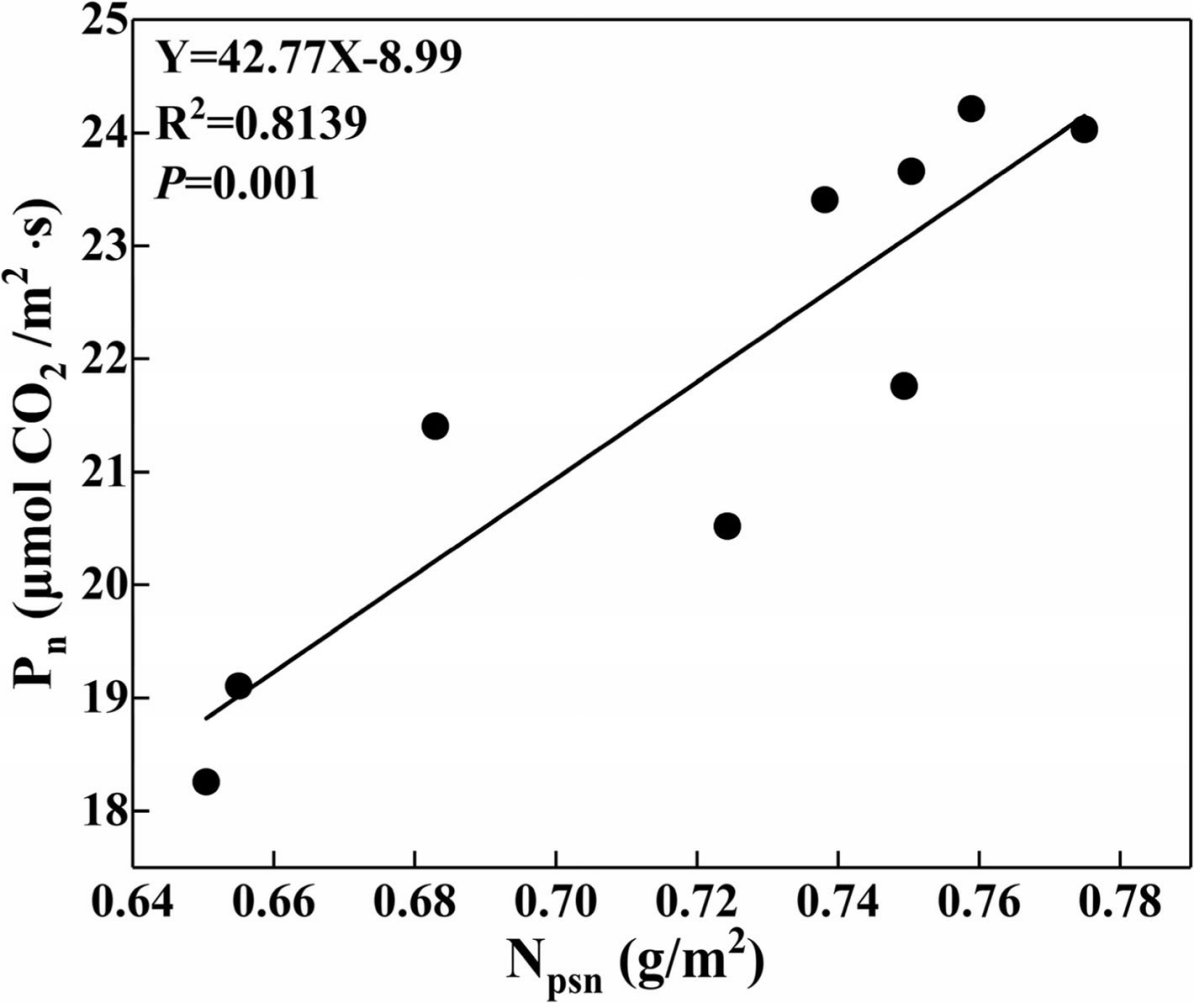
Relationship between the photosynthetic N (N_psn_) and photosynthetic rate (P_n_)

**Fig. 5:**
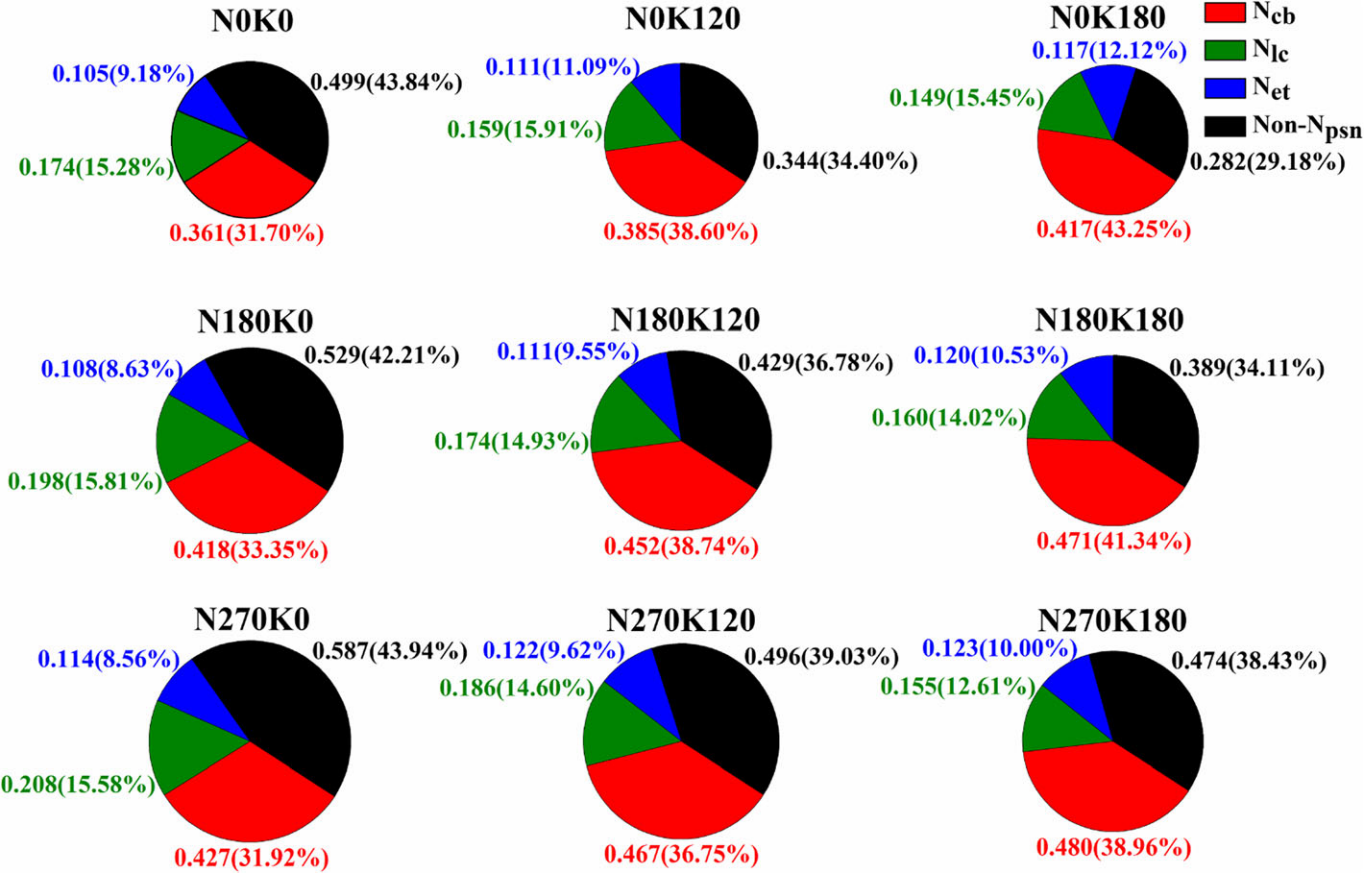
Interactive effects of nitrogen and potassium on N_psn_ allocation by function. N_cb_ represents carboxylation N, N_lc_ represents light capture N, N_et_ represents electron transfer N. Non-N_psn_ represents non-photosynthetic N. The number out of the bracket represents the absolute N content (g m^− 2^) of different functions. The number in the bracket represents the relative content (%)

There was high active relationship between the N_psn_ content and P_n_ (Fig. 4). Interactive effects of N and K on N_psn_ allocation were shown in Fig. 5. The results indicated that the increase of N rate significantly increased the absolute content (g m^− 2^) (outside of the bracket) of carboxylation N (N_cb_), light capture N (N_lc_), and Non-N_psn_, while decreased the absolute content of electron transfer N (N_et_). However, the relative content (%) (in the bracket) of N_cb_, N_lc_ and N_et_ was decreased with the increase of N rate, while that of Non-N_psn_ was increased with the increase of N rate. Compared with N0K0, N0K120 and N0K180, the supply of N increased the absolute content of N_cb_ by 17.0, 19.4 and 14.0%, and that of N_lc_ was increased by 16.7, 13.2 and 5.7%, and that of N_et_ was increased by 5.7, 5.0 and 3.8%, and that of Non-N_psn_ was increased by 11.8, 34.4 and 53.0% on average, respectively.

Both the absolute and relative content of N_cb_ and N_et_ were significantly increased with the supply of K. Compared with N0K0, N180K0 and N270K0, the supply of K increased the absolute content of N_cb_ by 11.1, 10.4 and 10.9% respectively, and the relative content of N_cb_ was increased by 29.1, 20.1 and 18.6% on average. The absolute content of N_et_ was increased by 8.6, 6.9 and 7.5%, and the relative content of N_et_ was increased by 26.4, 16.3 and 14.6% on average, respectively. Both the absolute and relative content of N_lc_ and Non-N_psn_ were significantly decreased with the supply of K. Compared with N0K0, N180K0 and N270K0, the supply of K decreased the absolute content of N_lc_ by 11.5, 15.7 and 18.0%, and the relative content of N_lc_ was decreased by 2.6, 8.6 and 12.7% on average, respectively. The absolute content of Non-N_psn_ was decreased by 37.3, 22.7 and 17.4%, and the relative content of N_et_ was decreased by 27.5, 16.0 and 11.9% on average, respectively.

**Fig. 6:**
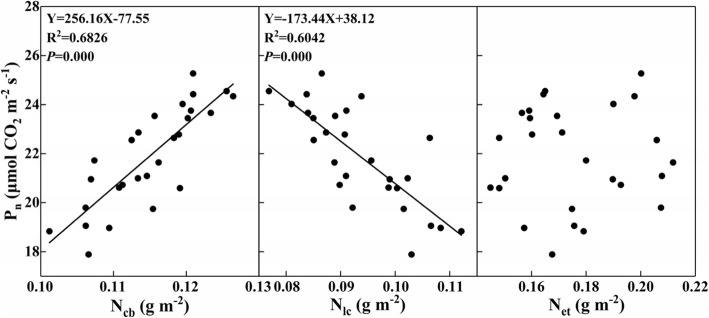
Relationship between the carboxylation N (N_cb_), light capture N (N_lc_) and electron transfer N (N_et_) with photosynthetic rate (P_n_)

The correlation analyses showed that were high active relationships between the N_cb_ and N_lc_ with P_n_ (Fig. 6).

### Photosynthetic N use efficiency

**Fig. 7:**
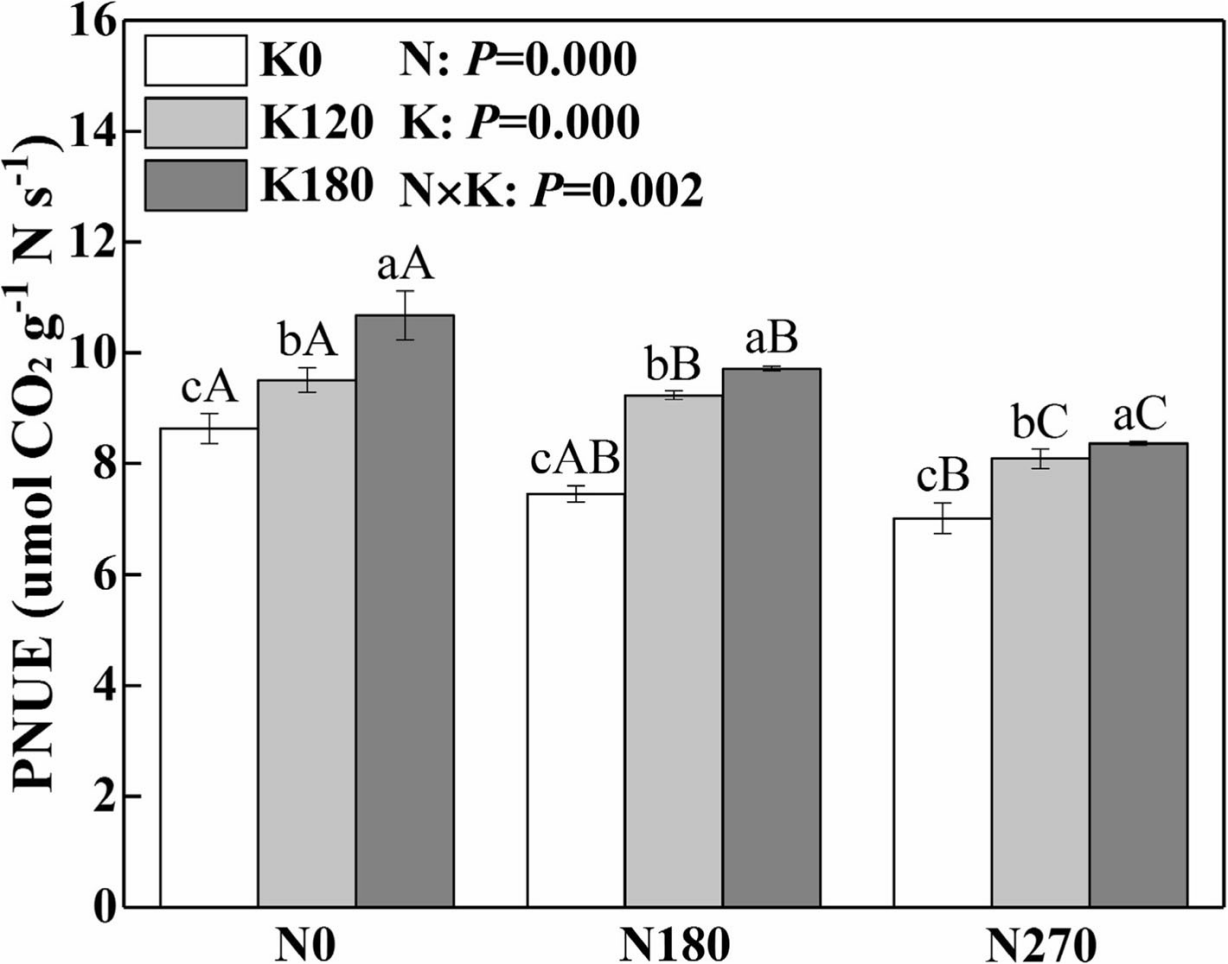
Interactive effects of nitrogen and potassium rates on photosynthetic N use efficiency (PNUE)

The supply of N and K had opposite effects on photosynthetic N use efficiency (PNUE) (Fig. 7). The PNUE decreased with the supply of N and increased with the supply of K. Compared with N0K0, N0K120 and N0K180, N supply under three K rates decreased the PNUE by 16.2, 8.9 and 15.3% on average, respectively. Compared with N0K0, N180K0 and N270K0, K supply under three N rates increased the PNUE by 16.9, 27.1 and 17.2% respectively.

## Discussion

As is well documented, the deficiency of N and K caused stunted leaf development and plant growth by reducing the P_n_ [9, 21–24]. The reduction in leaf area could maintain a relative high leaf N content and contribute to the maintenance of P_n_ [25, 26]. Under the experimental conditions of our study, the N and K deficiency caused a reduction of leaf dry mass by 43.3 and 22.1%, and leaf area was reduced by 77.9 and 36.6% respectively. The N_a_ was decreased by 21.8% for the deficiency of N but increased by 6.4% for the deficiency of K, which indicated that the supply of K could further decrease the N_a_. Previous studies also showed that the supply of K profoundly reduced the NH_4_^+^ cycling at the plasma membrane and diminished the excessive rates of unidirectional influx and efflux [27–29]. However, compared with the N0K0, N180K0 and N270K0, the supply of K increased the P_n_ by 9.9, 12.3 and 12.0% respectively. Based on our original assumption that the supply of K could influence the leaf N allocation, what happened to the leaf N allocation? Compared with N0K0, N180K0 and N270K0, the supply of K increased the relative content of N_psn_ by 17.7, 8.8 and 7.3%, while the relative content of N_store_ was decreased by 24.2, 11.4 and 8.7% on average, respectively. It could be clearly that the N_psn_ content was increased with the supply of K, even though the N_a_ was decreased. The increase of N_psn_ showed significant active correlation with P_n_ (Fig. 4). Previous studies had also reported the significant active correlations between N_psn_ with P_n_ [30, 31]. Under low N stress, leaf tended to invest more N into N_et_ to sustain the electron transport, which was consisting with the previous study on maize [32]. In contrast, high N promoted more N allocated to Rubisco and increased the Rubisco content, which was generally recognized as the most important reasons for the low PNUE under high N treatment [32–34]. In studies of physiological mechanisms of leaf PNUE, Rubisco has been focused upon for the key enzyme of photosynthesis and occupied much N in leaf [2]. It has been shown that species with a higher PNUE allocate more N to Rubisco [4, 35, 36]. Thus N allocation to photosynthetic proteins is one responsible reason for variation of PNUE. Supply of K increased leaf K content, but deceased the leaf N/K ratio, which had significant negative correlation with PNUE (Additional file 1: Figure S1). It indicated that the PNUE could be reduced with an appropriate reduction of leaf N/K, whether through decreasing N rate or increasing K rate.

As explained in the material method, N_store_ was defined as the N_a_ minus N_str_, N_rep_ and N_psn_. The N_store_ was assumed to be used in new tissues synthesis and metabolic enzymes photosynthesis products, which was including inorganic N, amino acid and some other proteins [17]. The same to stored carbohydrates, N_store_ could also promote plant growth and survival under reduced soil N availability [37]. Based on equation 10 and Additional file 2: Figure S2, we not only got that the total leaf N_store_ was decreased with the increase of K, but also clearly saw that the decreased N_store_ was resulted from the significantly decrease of N_np_.

The model used in this study helped us better understand the mechanism of N limitation upon leaf P_n_ and leaf N allocation with the interactive effects of N and K. The supply of N increased the leaf total N content and the absolute content of N_psn_ and N_store_. The relative content of N_store_ was increased while the N_psn_ was decreased with the supply of N, which means a greater proportion of leaf N was allocated to N_store_. The supply of K decreased the absolute and relative content of N_store_, but increased the absolute and relative content of N_psn_. There was high active relationship between the N_psn_ content and N_psn_ (Fig. 4), which indicated the importance of N_psn_ to N_psn_. Our findings highlights the need for further comparative research on the interaction of N and K, which could further improve the leaf P_n_, PNUE and grain yield of rice by optimizing leaf N allocation.

Indeed, the ear and sheath are also believed to play significant roles as sources of photosynthesis in addition to leaf [38–41]. Maize develops dimorphic chloroplasts which reside separately in sheath and mesophyll cells and cooperate to complete photosynthesis [42]. Chloroplast in sheath cells develop parallel lamellae with accumulated starch and diffuse grana, while the chloroplast in mesophyll cells develop thick grana [43]. In addition, the sheath and leaf showed different photosynthesis intensities for the different anatomical structures [41]. So, we assume that the mechanism of N and K on N allocation maybe different among different organs. Based on the results of this research, effects of N and K rates on sheath N allocation should be studied in future, which would be helpful to further understand the mechanism of N and K rates on crop photosynthesis. In addition to this, previous studies also showed that the N allocation was affected by methyl jasmonate [44, 45]. The methyl jasmonate reduced the expression of the chlorophyll biosynthesis genes like OsZIP1 and OsUPD2, down-regulated two chlorophyll biosynthesis-related proteins, four light-harvesting complex chlorophyll a/b-binding proteins and Rubisco small subunit, which indicated that the methyl jasmonate inhibited the photosynthesis via decreased of chlorophyll and photosynthesis-associated proteins [45]. However, the molecular mechanism of leaf N allocation under the interaction of N and K is still unclear.

## Conclusions

The present study demonstrated that N supply increased the N_a_, N_store_ and N_psn_ content, but decreased the relative content of N_psn_, which indicated more leaf N was allocated into non-photosynthetic N with the increase of N rates. K supply did decrease the N_a_ but increased the N_psn_ content and then increased leaf P_n_. The supply of K promoted the transformation of N_store_ to N_psn_ and increased the leaf PNUE. The decreased N_store_ mainly resulted from the decrease of non-protein N with the increase of K rates. From this new viewpoint of leaf N allocation, combined use of N and K could optimize the leaf N allocation and improve the N_psn_ content, and maintain relative high leaf P_n_ and PNUE. Taken together, these results contribute a further understanding of interaction between N and K, and the responses of leaf N allocation to different N and K rates.

## Methods

### Experiment site

The field trial was conducted from May to October in 2016 in Wuxue county (30°06′46″N, 115°36′9″E), Hubei province, central China. The experimental site was located in the subtropical monsoon climate zone, where the average temperature and total precipitation during the rice growing season was 27.7 °C and 1118.3 mm. The soil properties in 0–20 cm deep soil layer were as follows: pH 5.8 (soil/water = 1:2.5), 32.1 g kg^− 1^ organic matter, 1.8 g kg^− 1^ total N, 13.4 mg kg^− 1^ Olsen-P, 44.5 mg kg^− 1^ readily available K, and 302.5 mg kg^− 1^ slowly available K.

### Experiment design

It was a complete randomized block field experiment with three N and three K rates. Three N treatments were 0 (N0), 180 (N180), and 270 kg N ha^− 1^ (N270). Three K treatments were 0 (K0), 120 (K120), and 180 kg K_2_O ha^− 1^ (K180). The N fertilizer was applied in three doses: 50, 25 and 25% as transplanting (1 day before transplanting), tillering (8 days after transplanting) and booting (56 days after transplanting) fertilizer respectively. All treatments were supplied with 90 kg P_2_O_5_ ha^− 1^ and applied as transplanting fertilizer. The K fertilizer was applied in two doses: 75 and 25% as transplanting and booting fertilizer. Urea (46% N), superphosphate (12% P_2_O_5_) and potassium chloride (60% K_2_O) were used as the N, P and K fertilizer sources.

The area of each plot was 20 m^2^ (4 m × 5 m). All treatments were repeated three times in the field. All plots were plowed and leveled after the application of transplanting fertilizer.

### Crop cultivation

Seeds of *Oryza sativa* L. ssp. *japonica* (Shenliangyou 5814, Hunan Ava Seeds Co., Ltd., China) were sown on May 22 and transplanted on June 29 with the same hill space (24 cm × 15 cm). Soil bunds between different plots were covered with plastic film to prevent the exchange of water and fertilizer. All treatments received the same fungicides, insecticides and herbicides, and no obvious diseases, pests or weeds were present during the rice growing season. The grain yield was determined on October 1, 2016.

### Gas exchange measurement

The flag leaf gas exchange was measured using a Li-6400XT portable photosynthesis system (Li-Cor, Inc., USA) at stem elongation stage (52 days after transplanting). The cuvette conditions were 1200 μmol m^− 2^ s^− 1^ photosynthetic photon flux density, 400 μmol m^− 2^ s^− 1^ CO_2_ in the leaf chamber (C_a_), 500 μmol s^− 1^ flow rate, 60% relative humidity, and 30.0 °C of the temperature respectively.

The P_n_ − C_i_ (C_i_ is the CO_2_ concentration in leaf internal air space) curves were measured. The C_a_ was set stepwise from 400 to 300, 200, 100, 50, and thento 400, 600, 800, 1000, 1200 and 1500 μmol mol^− 1^. The maximum rate of Rubisco-catalyzed carboxylation rate (V_c,max_) and the maximum rate of electron transport rate (J_max_) were calculated as defined by Long and Bernacchi [46].

### Leaf sampling

Leaf discs were created with a 5 mm diameter punch (the midvein was avoided) after the measurement of photosynthesis. Ten discs were weighed and stored at − 80 °C. The rest of leaf samples were desiccated at 105 °C for 0.5 h and then dried at 65 °C to constant weight. Three representative hills were sampled from each plot directly at the same time. The leaves of each plant were cut off, and the leaf area was measured using Image-Pro Plus 6.0 software (Media Cybernetics, Silver Spring, MD, USA).

### Nitrogen allocation by morphology

**Fig. 8:**
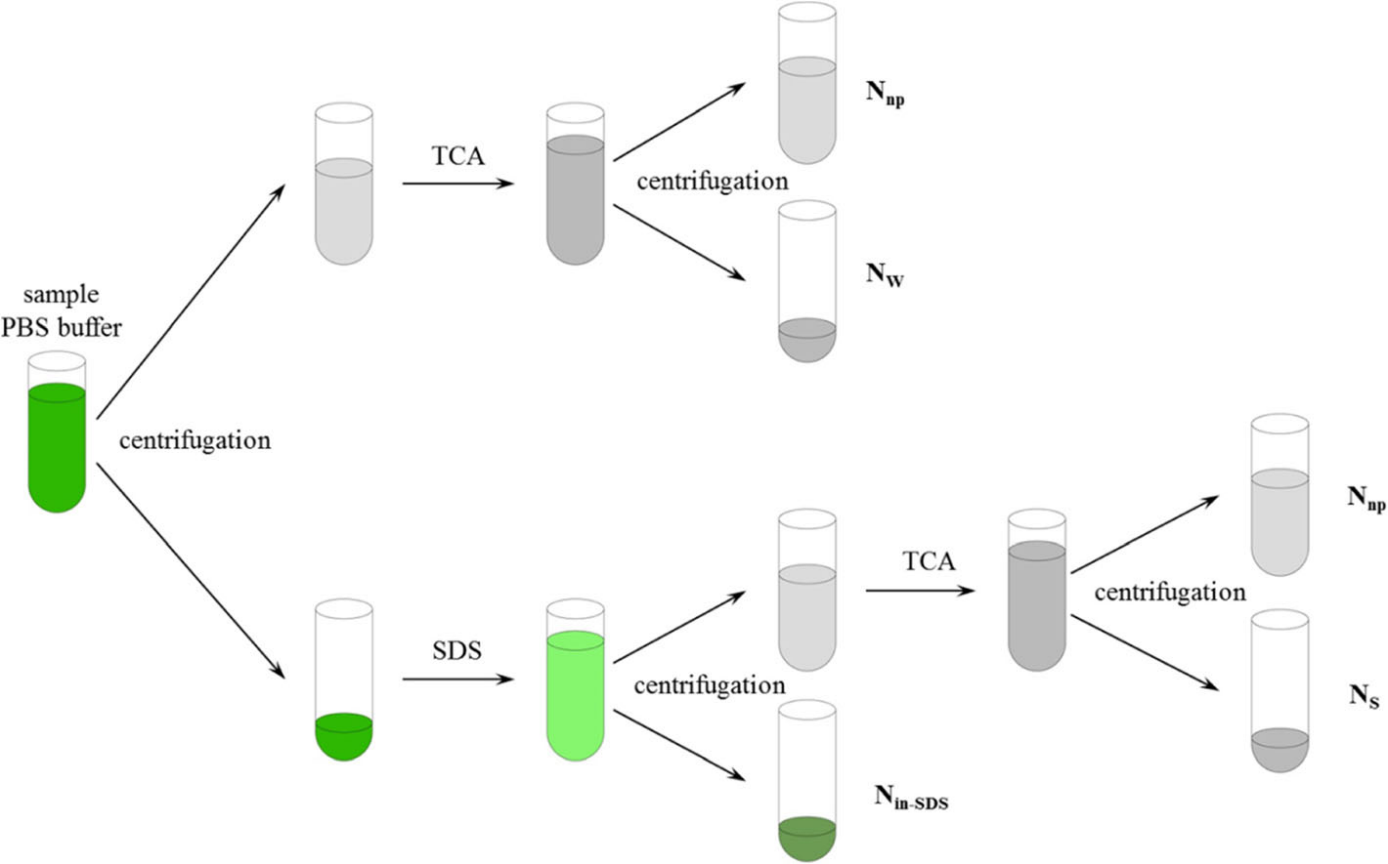
Flow chart for the extraction of different N morphology. PBS (Na phosphate buffer). TCA (20% trichloroacetic acid), SDS (3% sodium dodecyl sulfate)

**Fig. 9:**
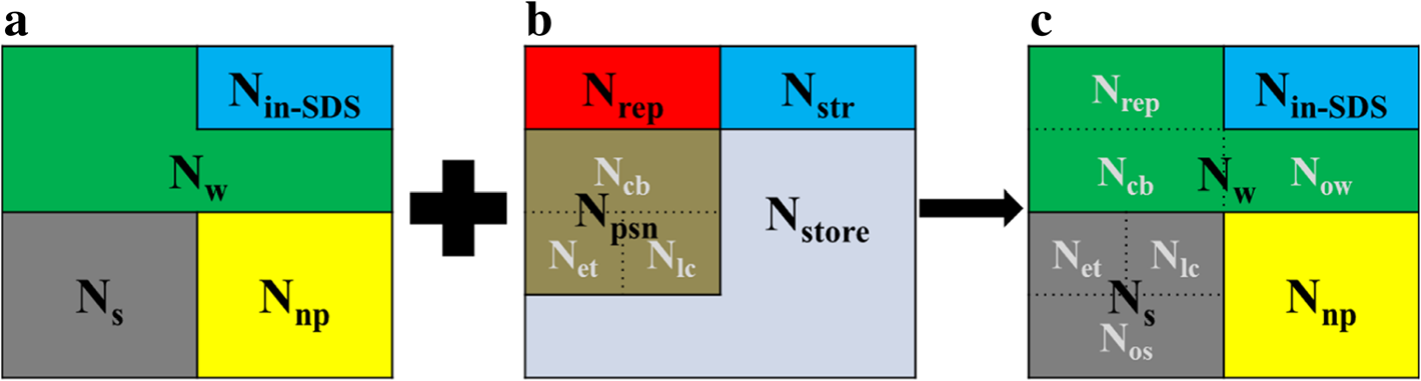
New model for distinguishing different leaf N by N morphology and N function. Leaf N was divided into water-soluble protein N (N_w_), SDS-soluble protein N (N_s_), SDS-insoluble protein N (N_in-SDS_) and non-protein N (N_np_) by morphology (**a**). Leaf N was divided into respiratory N (N_rep_), structural N (N_str_), photosynthetic N (N_psn_, which was composed by carboxylation N (N_cb_), electron transfer N (N_et_) and light capture N (N_lc_)) and storage N (N_store_) by function (**b**)

The frozen leaf discs were used to measure the different forms of N (Fig. 8) [31]. Leaf samples were powdered in liquid N using a chilled mortar and pestle and homogenized with 1 ml of 100 mM phosphate buffer saline (PBS, pH was 7.5) and decanted it into a 10 ml centrifuge tube. The pestle and mortar were washed with 1 ml phosphate buffer and decanted into the same centrifuge tube, and repeated that for four times. The supernatant (water-soluble protein, N_w_) was separated after a 15-min centrifugation at 15000 *g* and 4 °C. 1 ml of phosphate buffer containing 3% sodium dodecyl sulfate (SDS) was added to the centrifuge tube with leaf sample and heat up for five minutes in constant temperature water at 90 °C. The supernatants (SDS-soluble protein, N_s_) was collected in a new centrifuge tube after a 10-min centrifugation at 4500 *g* and repeated that for six times. The residual sample (SDS-insoluble protein, N_in-SDS_) was washed by ethanol and filtered with quantitative paper. Equal volume of 20% trichloroacetic acid (TCA) was added to the supernatant to denature the protein. The precipitate was filtered with quantitative paper and washed by ethanol. Three kinds of N were dried and digested with H_2_SO_4_-H_2_O_2_, and the same to the dried leaf discs, which was used to determine the leaf total N [47]. Non-protein N (N_np_) in leaf (inorganic N and N-containing small molecules like amino acids) was the leaf totals N subtract the upper three kinds of N (Fig. 9).

### Nitrogen allocation by function

According to the mechanistic model of leaf utilization of N for assimilation [17, 48], leaf N consisted of photosynthetic N (N_psn_), respiration N (N_resp_, respiratory enzymes located in mitochondrial matrix), structural N (N_str_, used to build cell walls) and storage N (N_store_, N stored in plant tissues but not participated in any metabolic processes or structural components) (Fig. 9). The N_psn_ could also be divided into three different parts: proteins for carboxylation in the Calvin cycle (N_cb_), proteins for light capture in PSI, PSII, and other light-harvesting pigment protein complexes (N_lc_), and proteins involved in electron transport (N_et_). Here are the calculation equations for different kinds of N in leaf.

1 $$ {\mathrm{N}}_{\mathrm{psn}}={\mathrm{N}}_{\mathrm{cb}}+{\mathrm{N}}_{\mathrm{lc}}+{\mathrm{N}}_{\mathrm{et}} $$

2 $$ \mathrm{Ncb}=\frac{\mathrm{Vc},\max }{6.25\times \mathrm{Vcr}\times {\mathrm{f}}_{\mathrm{Vc},\max }\ } $$

where 6.25 is the N conversion coefficient into Rubisco (g Rubisco g^− 1^ leaf N) [49], V_cr_ is 20.8 umol CO_2_ g^− 1^ Rubisco s^− 1^ at 25 °C [50], f_Vc,max_ is a correction coefficient for V_c,max_ and it is 0.361 at 35 °C.

3 $$ \mathrm{Nlc}=\frac{\mathrm{Cchl}}{\mathrm{CB}} $$

where C_chl_ is the chlorophyll content (mmol m^− 2^), C_B_ is the ratio of chlorophyll to organic N in light harvesting components (2.15 mmol g^− 1^) [50].

4 $$ \mathrm{Net}=\frac{\mathrm{Jmax}}{8.06\times \mathrm{Jmc}\times {\mathrm{f}}_{\mathrm{Jmax}}} $$

where 8.06 is the N binding coefficient for cytochrome f [51], J_mc_ is 155.65 umol e^−^ umol cytochrome f s^− 1^ at 25 °C [50], fJ_max_ is a correction coefficient for J_max_ and it is 0.524 at 35 °C.

5 $$ \mathrm{Nresp}=\frac{\mathrm{Rt}}{33.69\times {\mathrm{f}}_{\mathrm{r}}} $$

6 $$ {\mathrm{R}}_{\mathrm{t}}=0.015\ {\mathrm{V}}_{\mathrm{c},\max } $$

where R_t_ is the leaf respiration rate (μmol CO_2_ m^− 2^ s^− 1^) that calculated based on V_c, max_ [52], 33.69 is the leaf N use efficiency for respiration at 25 °C (μmol CO_2_ g^− 1^ N s^− 1^) [3], f_r_ is the respiration correction coefficient and it is 0.561.

The remaining fraction after the remove of N_psn_, N_resp_ and N_str_ of total leaf N content (N_a_) is the N_store_.

7 $$ {\mathrm{N}}_{\mathrm{store}}={\mathrm{N}}_{\mathrm{a}}-{\mathrm{N}}_{\mathrm{psn}}-{\mathrm{N}}_{\mathrm{resp}}-{\mathrm{N}}_{\mathrm{str}} $$

where N_a_ is the total leaf N content, N_str_ is the structural N (which is also the SDS-insoluble N, N_in-SDS_).

The remaining N_w_ without N_cb_ and N_resp_ could be regarded as the other water-soluble protein (N_ow_) of N_store_.

8 $$ {\mathrm{N}}_{\mathrm{ow}}={\mathrm{N}}_{\mathrm{w}}-{\mathrm{N}}_{\mathrm{cb}}-{\mathrm{N}}_{\mathrm{resp}} $$

The remaining N_s_ without N_lc_ and N_et_ could be regarded as the other SDS-soluble protein (N_os_) of N_store_.

9 $$ {\mathrm{N}}_{\mathrm{os}}={\mathrm{N}}_{\mathrm{s}}-{\mathrm{N}}_{\mathrm{lc}}-{\mathrm{N}}_{\mathrm{et}} $$

Based on the model (Fig. 9), the N_store_ then could be expressed in another way:

10 $$ {\mathrm{N}}_{\mathrm{store}}={\mathrm{N}}_{\mathrm{os}}+{\mathrm{N}}_{\mathrm{ow}}+{\mathrm{N}}_{\mathrm{np}} $$

where N_np_ is other non-protein N of N_store_.

### Nitrogen and potassium measurement

The N concentration in the digestion solution was determined with a continuous flow analyzer (AA3, Seal Analytical, Inc., Southampton, UK), and the K concentration in the digestion solution was determined with a flame photometer (M-410, Cole-Parmer, Chicago, IL, USA.).

### Statistical analysis

An analysis of variance (ANOVA) was calculated using SPSS 18.0 to compare the differences between different treatments. Differences between the mean values were compared using Duncan’s multiple range test at a *P* ≤ 0.05 level of significance. All figures and regression analyses were created using Origin Pro 8.5 software.

## Additional files

Additional file 1: Relationship between the leaf N/K ratio and photosynthetic N use efficiency (PNUE). (DOCX 151 kb)

Additional file 2: Interactive effects of nitrogen and potassium rates on other SDS-soluble protein (N_os_), other water-soluble protein (N_ow_) and non-protein N (N_np_). (DOCX 239 kb)

## Data Availability

The datasets used and analyzed during the current study are available from the corresponding author on reasonable request.

## Abbreviations

C_a_: CO_2_ in the leaf chamber
C_i_,: CO_2_ concentration in leaf internal air space
J_max_: The maximum rate of electron transport rate
K: Potassium
K0: The treatment with 0 kg K_2_O ha^− 1^
K120: The treatment with 120 kg K_2_O ha^− 1^
K180: The treatment with 180 kg K_2_O ha^− 1^
K_a_: K content
N: Nitrogen
N0: The treatment with 0 kg N ha^− 1^
N180: The treatment with 180 kg N ha^− 1^
N270: The treatment with 270 kg N ha^− 1^
N_a_: N content
N_cb_: Carboxylation N
N_et_: Electron transfer N
N_in-SDS_: SDS-insoluble protein N
N_lc_: Light capture N
N_np_: Non-protein N
N_os_: Other SDS-soluble protein
N_ow_: Other water-soluble protein
N_psn_: Photosynthetic N
N_rep_: Respiratory N
N_s_: SDS-soluble protein N
N_store_: Storage N
N_str_: Structural N
N_w_: Water-soluble protein N
P_n_: Net photosynthetic rate
PNUE: Photosynthetic N use efficiency
SDS: Sodium dodecyl sulfate
TCA: Trichloroacetic acid
V_c,max_: The maximum rate of Rubisco-catalyzed carboxylation rate
